# Laparoscopic-Assisted Recipient Nephrectomy and Recipient Kidney Procurement during Orthotopic Living-Related Kidney Transplantation

**DOI:** 10.1155/2011/153493

**Published:** 2011-07-28

**Authors:** Dimitri Mikhalski, Karl Martin Wissing, Renaud Bollens, Daniel Abramowicz, Vincent Donckier, Anh-Dung Hoang

**Affiliations:** ^1^Department of Digestive Surgery and Transplantation, Hôpital Erasme, Université Libre de Bruxelles, 1070 Brussels, Belgium; ^2^Department of Nephrology, Universitair Ziekenhuis Brussel, 1090 Brussel, Belgium; ^3^Department of Urology, Hopital de la Dorcas, 7500 Tournai, Belgium; ^4^Department of Nephrology, Hôpital Erasme, Université Libre de Bruxelles, 1070 Brussels, Belgium

## Abstract

Advanced atherosclerosis or thrombosis of iliac vessels can constitute an absolute contraindication for heterotopic kidney transplantation. We report the case of a 42-year-old women with end-stage renal disease due to lupus nephritis and a history of bilateral thrombosis of iliac arteries caused by antiphospholipid antibodies. Occlusion had been treated by the bilateral placement of wall stents which precluded vascular anastomosis. The patient was transplanted with a right kidney procured by laparoscopic nephrectomy from her HLA semi-identical sister. The recipient had left nephrectomy after laparoscopical transperitoneal dissection. The donor kidney was orthotopically transplanted with end-to-end anastomosis of graft vessels to native renal vessels and of the graft and native ureter. Although, the patient received full anticoagulation because of a cardiac valve and antiphospholipid antibodies, she had no postoperative complication in spite of a short period of delayed graft function. Serum creatinine levels three months after transplantation were at 1.0 mg/dl. Our case documents that orthotopical transplantation of laparoscopically procured living donor kidneys at the site of recipient nephrectomy is a feasible procedure in patients with surgical contraindication of standard heterotopic kidney transplantation.

## 1. Case Report

We report the case of a 42-year-old woman who underwent orthotopic renal transplantation for the first time, using a kidney from her sister. The patient had developed end-stage renal disease secondary to lupus nephritis. She had a medical history of bilateral thrombosis of both common and external iliac arteries due to lupus anticoagulant and antiphospholipid antibodies that had required bilateral percutaneous angioplasty with wall stenting. The medical workup had documented thrombosis of both iliac veins and of the lower portion of the lower part of the inferior vena cava as well as extensive atherosclerotic lesions of the abdominal aorta. In addition, she had a history of mitral valve replacement with a St. Jude prosthetic valve and received anticoagulation therapy with acenocoumarol. 

**Figure 1:**
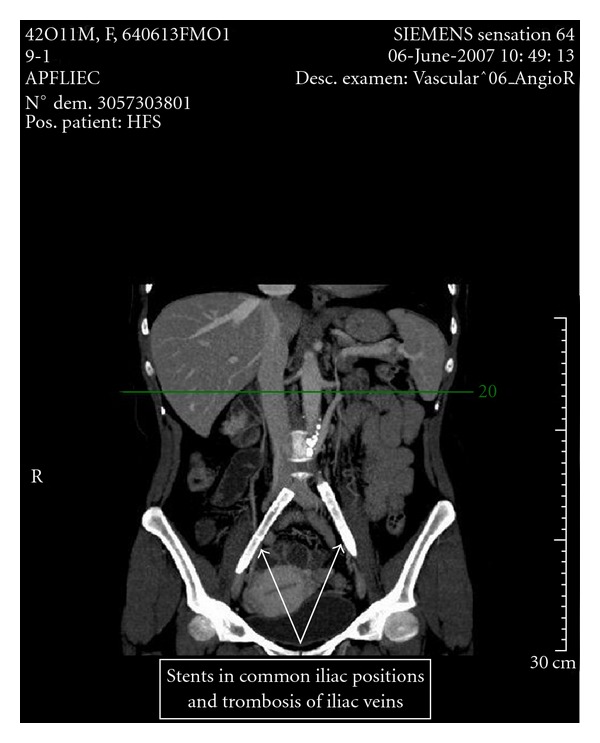
CT angiography image of the receiver pelvic vessels.

**Figure 2:**
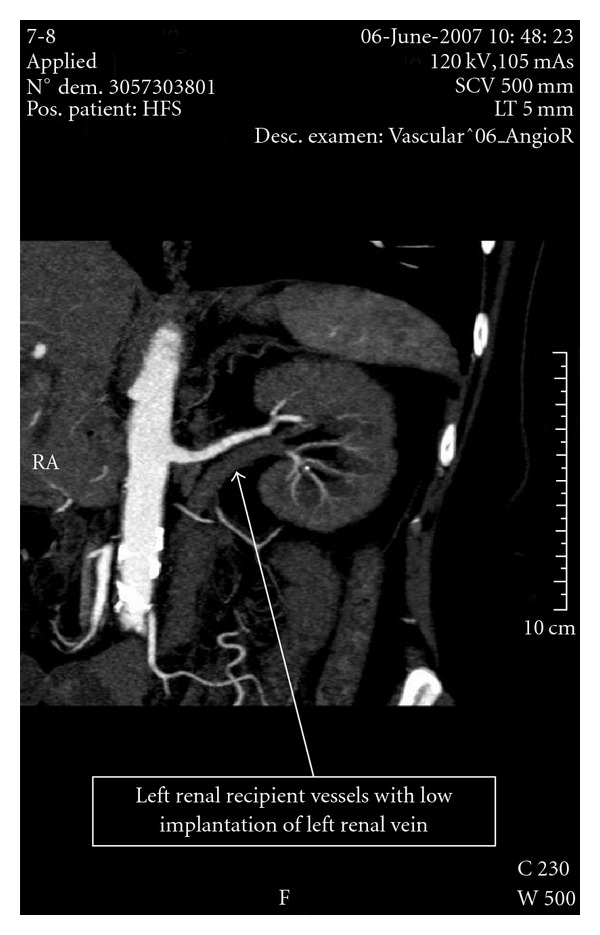
CT angiography image of the receiver left renal vessels.

The patient had an HLA semi-identical sister who was willing to donate a kidney. The donor workup had documented a slightly lower tubular mass of the right kidney which was chosen for procurement. The “classic” extraperitoneal pelvic transplantation of a kidney graft was contraindicated because of stented iliac arteries and extended atheromatosis of the aorta (Figure 1). An alternative surgical technique with the transplantation of the graft into the location of a native kidney was proposed to the recipient and her sister who both consented to the procedure. Mapping CT-angiography of the recipient showed that the left renal artery was appropriated for anastomosis with the graft artery. At the same time the anatomical variant of the left recipient renal vein was found with low implantation to vein cava inferior (VCI; Figure 2). 

The graft was procured by laparoscopic right donor transperitoneal nephrectomy with utilization of linear stapler Endo-GIA as previously described [1]. The warm ischemic time was 3 minutes measured from the first clip applied to the renal artery until the kidney was perfused with preservation solution. The graft was cold flushed with HTK solution and preserved on ice during the left nephrectomy of the recipient. The total duration of the donor nephrectomy was 95 minutes and total blood loss due to the procedure was 25 mL. The donor was discharged at day 4 after the nephrectomy.

The recipient was placed in a modified lateral decubitus position and Table 1 flexed for hyperextension of the left flank. Pneumoperitoneum was established via a Veress needle placed two fingerbreaths below the left costal margin, at the level of the lateral border of the rectus muscle, which was replaced by a 10 mm port for a 0-degree lens optic. Under direct vision the 10 mm trocar for a bipolar grasper, two 5 mm trocar for the monopolar scissors and for a suction device were inserted. The descending colon and the spleen were dissected from the underlying Gerota's fascia. Following the medial mobilization of the colon and the mesocolon, the gonadal vessels were visualized and preserved during operation. The fatty tissue at the level of the lower pole of the kidney was incised and lifted to locate the psoas muscle. By tracking the cephalic course of the ureter, the plane was followed up to the renal pedicle. The left renal vein is dissected and gently displaced till its low implantation in VCI to expose the required length and visualize lumbar and adrenal branches. At this level, the left renal artery was dissected and exposed from its origin to the first bifurcation. The adrenal gland was preserved and separated from the kidney. The posterior and lateral attachments of the kidney to the abdominal wall are released by blunt and sharp dissection. Then two trocar ports under the left costal margin were joined by a 2.5 inch (6 cm) incision to access the left recipient kidney. The kidney was attached only to the hilum. The artery and the vein were clamped using bulldog clamps and the pedicle was divided close to the hilum. The ureter was sectioned at the level of the pyeloureteral junction and the kidney was removed. 

The native renal vessels were found to be sufficient and were used for the end-to-end anastomoses with prolene 6/00 for the artery and prolene 5/00 for the vein.

The left native ureter was then spatulated and anastomosed in an end-to-end fashion to the transplant ureter with vicryl 3–0, over double J stent. A lombonephropexy of the graft was performed and a silicone Jackson-Pratt drain was inserted. The abdominal wall was closed using running vicryl 2–0 suture for the peritoneum, interrupted vicryl 0 suture for the muscle, and running vicryl 1 for the aponeurosis, and the skin incisions were closed with intradermal suture. The second warm ischemia time was 17 minutes and blood loss in the recipient was 15 mL. 

**Figure 3:**
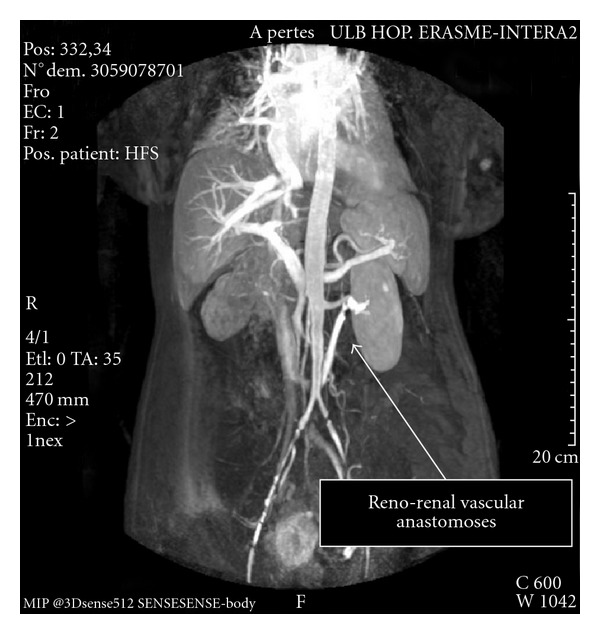
MRI angiography image after kidney transplantation in orthotopic position.

At the moment of transplantation, the patient had maintenance immunosuppression with azathioprine and steroids for her systemic lupus erythematosus. Basiliximab and tacrolimus were added for prevention of acute graft rejection. The patient had delayed graft function up to day 4 but did not require renal replacement therapy because of the residual function of her native kidney. Anticoagulation consisted first of low molecular weight heparine (enoxaparin 20 mg per day) with resumption of acenocoumarol on postoperative day 6. The only medical complication was a urinary infection with Pseudomonas Aeruginosa treated with ciprofloxacine. The patient left the department two weeks after transplantation with a serum creatinine of 1.2 mg/dL. The double-J stent was removed 4 weeks after transplantation. Three months after transplantation a control angio-MRI showed normal a normal kidney and graft vessels (Figure 3). At this moment the serum creatinine level and glomerular filtration rate were 1.0 mg/dL and 58 mL/min/1.73^2^, respectively.

## 2. Discussion

We present a first case of orthotopic transplantation of a living-donor kidney using laparoscopic techniques for nephrectomy of both the donor and recipient kidneys in young patients with complex vascular disease contraindicating classical heterotopic transplantation. Our case demonstrates that these surgical procedures permit successful renal transplantation in selected candidates that would otherwise be forced undergo life-long renal replacement therapy.

We chose laparoscopic donor nephrectomy as these technique better aesthetic outcomes, a shortened hospital stay and overall better quality of life as compared to the open procedure while resulting in equivalent graft outcomes [2]. In addition, we have developed an original procurement technique in our center which allows safe and rapid right nephrectomy with adequate length of donor vessels [1]. This technique has also been used in the present procedure. The anastomosis of graft vessels in our patient to the lower abdominal aorta and vena cava was impossible due to extensive atheromatosis and venous thrombosis. Under these conditions an orthotopic kidney transplantation with anastomosing donor and recipient native vessels is well established [3–5] and had been previously performed in patients with an aortic occlusion, severe pelvic atherosclerosis, pelvic vascular anomaly, previews kidney transplantation, and urinary tract reconstruction [6]. 

We chose a laparoscopic technique for recipient nephrectomy to minimize surgical trauma and the risk of bleeding in this patient treated with chronic anticoagulation because of a metallic prosthetic valve and taking in the consideration that the postoperative complications after classic lumbotomy or mini-incision open donor nephrectomy described in 12% of cases [7, 8]. The technique was also used because it allows gentle dissection of the vascular structures and of the ureter without destruction of the surround tissue during nephrectomy and the mobilization of full length of renal vessels to facilitate the subsequent anastomosis of graft vessels and ureter.

In summary our case shows that living donor orthotopic kidney transplantation with laparoscopic donor and recipient nephrectomy is a feasible procedure associated with low morbidity and excellent graft outcome that can allow successful transplantation in patients with surgical contra-indications to classical heterotopic transplantation.

## Figures and Tables

**Table 1:** Donor and recipient data.

	Donor	Recipient
Age (years)	39	42
Sex	Female	Female
BMI	25.4	26.2
Side of nephrectomy	Right	Left
Blood loss	25 cc	15 cc
Operation time (skin-to-skin)	1 h 35 min	1 h 45 min
Hospital stay	4 days	13 days
day of the transplantation		
(i) creatinine level (mg/dL)	0.6	3.3
(ii) glomerular filtration (mL/min/1.73^2^)	>90	15
2 weeks after transplantation		
(i) creatinine level (mg/dL)	1.1	1.2
(ii) glomerular filtration (mL/min/1.73^2^)	68	51
1 year after transplantation		
(i) creatinine level (mg/dL)	0.9	1.1
(ii) glomerular filtration (mL/min/1.73^2^)	74	56
